# Metachronous liver metastases after long-term follow-up of endoscopic resection for rectal neuroendocrine neoplasms: a report of three cases

**DOI:** 10.1186/s40792-020-0792-5

**Published:** 2020-01-15

**Authors:** Yuma Hane, Takahiro Tsuchikawa, Kimitaka Tanaka, Yoshitsugu Nakanishi, Toshimichi Asano, Takehiro Noji, Yo Kurashima, Yuma Ebihara, Soichi Murakami, Toru Nakamura, Keisuke Okamura, Satoshi Takeuchi, Toshiaki Shichinohe, Satoshi Hirano

**Affiliations:** 1 Department of Gastroenterological Surgery II, Division of Surgery, Faculty of Medicine, Hokkaido University, West-7, North-15, Kita-ku, Sapporo, 060-8638 Japan; 2 Department of Medical Oncology, Graduate School of Medicine, Hokkaido University, Sapporo, Japan

**Keywords:** Neuroendocrine neoplasm, Endoscopic resection, Long-term follow-up, Recurrence

## Abstract

**Background:**

Rectal neuroendocrine neoplasms (NENs) are rare, but their incidence has increased in recent years. The metastasis rate is low in cases of a tumor diameter < 1 cm or depth of invasion lower than the submucosa; therefore, the European Neuroendocrine Tumor Society (ENETS) and the North American Neuroendocrine Tumor Society (NANETS) consensus guidelines recommend endoscopic resection. Since little has been reported on the long-term prognosis of endoscopic resection for rectal NEN, consensus is lacking regarding the follow-up period after endoscopic resection.

**Case presentation:**

Here, we report three cases of metachronous liver metastasis after long-term follow-up of endoscopic mucosal resection (EMR) for rectal NEN. The pathological findings indicated a depth lower than the submucosa and complete radical resection in all cases and lymphovascular invasion in only one case. All three cases showed metachronous multiple liver metastases after 9–13 years of follow-up for EMR, despite achieving complete resection and without muscular invasion.

**Conclusions:**

Metachronous liver metastases may occur after long interval following endoscopic resection; thus, long-term follow-up is necessary after endoscopic resection for rectal NEN.

## Background

Rectal neuroendocrine neoplasm (NEN) is a rare tumor derived from enterochromaffin cells with a reported incidence of 1.04 per 100,000 [1] that has recently been increasing. With the spread of chance to take the screening endoscopes, 93.3–100% are diagnosed at 1 cm or less [2]. NEN is described as a low-grade malignant tumor according to World Health Organization classification; however, the prognosis in cases of lymph node and distant metastasis is as poor as that of adenocarcinoma [3]. The metastasis rate at diagnosis is low in 3–9.7% of tumors measuring < 10 mm [4, 5]; therefore, the European Neuroendocrine Tumor Society (ENETS), North American Neuroendocrine Tumor Society (NANETS), and Japanese Neuroendocrine Tumor Society (JNETS) guidelines recommend endoscopic resection in cases of tumors < 10 mm without muscular invasion [6, 7]. Moreover, the metastasis rate at diagnosis is high in 56.7–73% of tumors measuring > 20 mm [4, 5], and surgical resection with prophylactic lymph node dissection is recommended as with colorectal carcinoma. On the other hand, endoscopic resection for intermediate tumors measuring 10–20 mm is considered as expanded indication despite little evidence [4, 8].

Collectively, in terms of the treatment options for rectal NEN < 20 mm, there are several options for endoscopic resection, including additional surgical resection with prophylactic lymph node dissection and appropriate follow-up periods, depending on the global guidelines due to little information about and evidence of long-term prognosis after endoscopic resection for rectal NEN. Here, we report three cases of metachronous liver metastasis after long-term follow-up of endoscopic mucosal resection (EMR) and discuss the current problems underlying the treatment options for small rectal NEN with a review of the literature.

## Case presentation

### Case 1

**Fig. 1:**
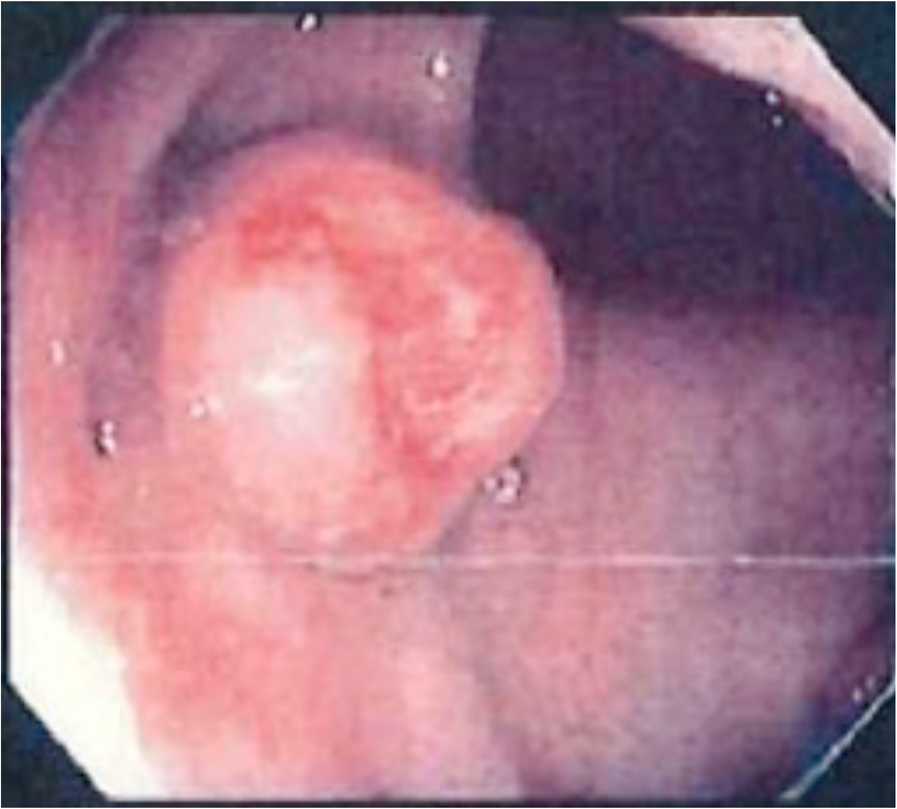
Endoscopy image of the primary tumor in case 1. Submucosal tumor with ulcer in the rectum

**Fig. 2:**
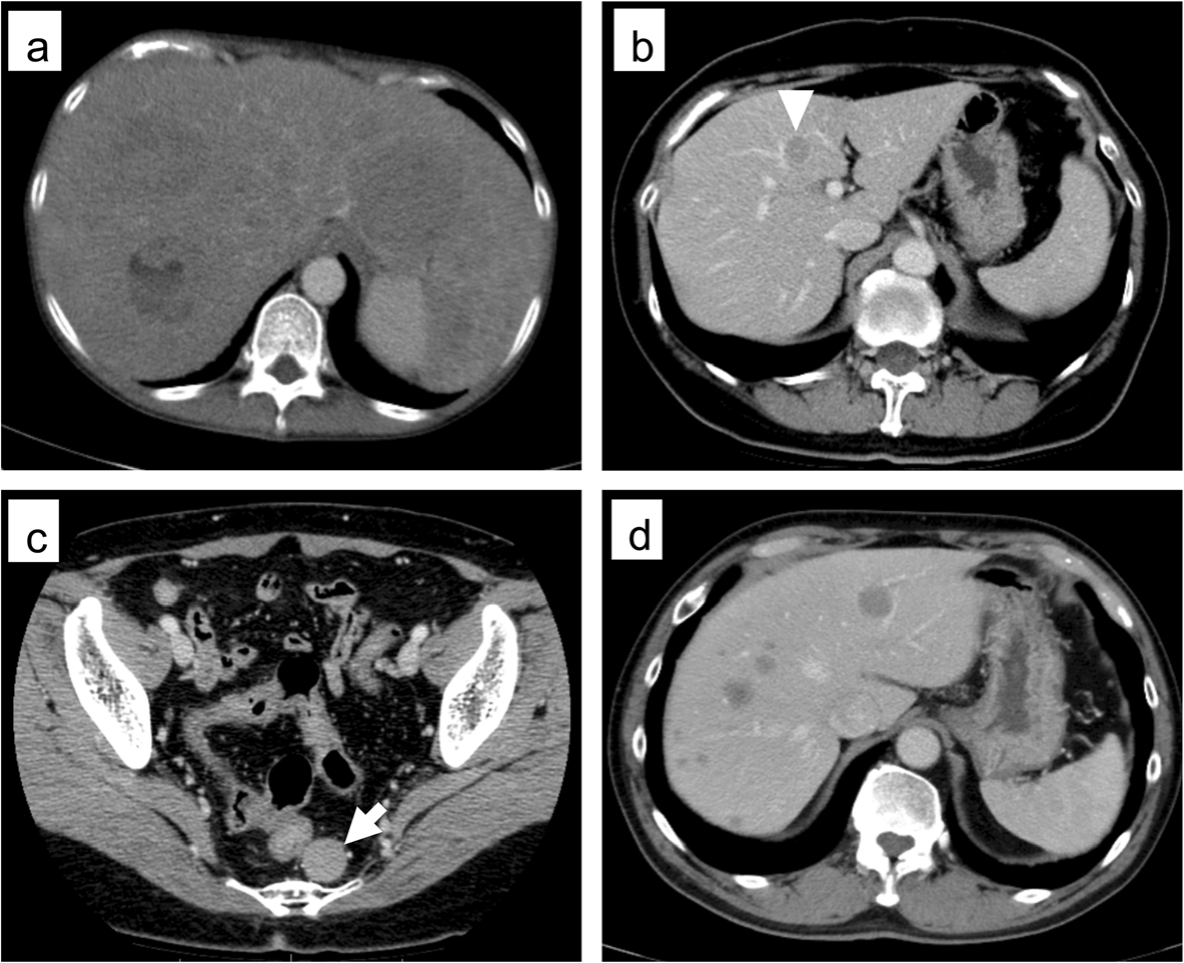
Selected images from abdominal computer tomography. **a** Case 1: multiple low-density lobular lesions in the bilateral lobe. **b** Case 2: low-density lesion in S4 (arrowhead). **c** Case 2: left dorsal recurrent mass of the rectum (arrow). **d** Case 3: multiple low-density lesions in the bilateral lobe

A 55-year-old woman underwent EMR for rectal NEN (Fig. 1). The pathological findings were as follows: well-differentiated NEN, tumor size 13 mm, no muscular invasion (submucosa), negative resection margins, no lymphovascular invasion, and Ki-67 < 1%. She underwent colonoscopy at 1, 2, 3, 6, and 9 years after EMR for rectal NEN, but no recurrence was revealed. At 10 years after EMR, she visited our hospital complaining of bloating, weight loss, and leg edema. Abdominal computed tomography (CT) revealed multiple liver masses in the bilateral lobe (Fig. 2a) diagnosed as multiple liver metastases by biopsy concordant with the initial tumor. Since immunohistochemistry staining of the liver metastasis showed somatostatin-2 receptor (SSTR-2) positivity, monthly intramuscular octreotide 30 mg was administered. The symptoms got worse, and daily oral everolimus 10 mg was added 4 months after the initial treatment. However, she developed pneumonia 3 weeks after the start of everolimus. Everolimus was changed to weekly streptozocin 1000 mg. Stable disease (SD) was achieved and maintained for 6 months after the initial treatment; however, it was discontinued due to liver failure. She died of liver metastasis 1 year after the initial treatment.

### Case 2

A 59-year-old woman underwent EMR for rectal NEN. The pathological findings were as follows: well-differentiated NEN, tumor size 10 mm, no muscular invasion (submucosa), negative resection margin, no lymphovascular invasion, and Ki-67 = 5%. A comprehensive medical checkup performed 9 years after EMR revealed two liver masses derived from S4 (15 mm) and S6/7 (15 mm) as well as a pelvic mass (18 mm) on CT (Fig. 2b, c). No tumors had infiltrated the adjacent tissues. Fine needle aspiration (FNA) and liver biopsy of the S4 liver mass revealed local recurrence and multiple liver metastases. She previously underwent laparoscopic low anterior resection (LAR) of the local recurrence in a prior hospital and radiofrequency ablation (RFA) for liver metastases. Pathological findings of pelvic mass resected by LAR revealed SSTR-2 positivity; therefore, she was started on monthly intramuscular octreotide 30 mg and was followed up for 3 months. However, CT revealed new liver metastasis in S3 at 1 year after the initial treatment for which she underwent RFA. Daily oral everolimus 10 mg was added. Since the liver metastasis was again enlarged 2 years after the initial treatment, weekly streptozocin 1000 mg administration was started. However, CT revealed enlargement of multiple liver metastases at 3 months after the first streptozocin administration; therefore, transhepatic arterial chemoembolization was performed. She has maintained stable disease (SD) for 3 years from the diagnosis of recurrence.

### Case 3

A 54-year-old man underwent EMR for rectal NEN. The pathological findings were as follows: well-differentiated NEN, tumor size 12 mm, no muscular invasion (submucosa), negative resection margins, positive vascular invasion, chromogranin positivity, synaptophysin positivity, and Ki-67 = 1.7%. He visited a nearby hospital complaining of right epigastric pain 13 years after the EMR. It was diagnosed as intraductal papillary mucinous neoplasm and pancreatitis. CT on admission showed multiple liver masses (Fig. 2d). A liver biopsy revealed multiple liver metastases derived from S6 (47 mm). He received daily oral everolimus 10 mg and has maintained SD for 3 years and 3 months since the diagnosis of liver metastases.

## Discussion

In this report, we showed three cases of metachronous liver metastasis after long-term follow-up of EMR for rectal NEN. For each case, EMR was performed according to the expanded indication of endoscopic resection in the ENETS and NANETS guidelines. Complete curative resection was achieved in all three cases; therefore, additional resection did not perform. To our knowledge, this is the first report of metachronous liver metastases after the long-term follow-up of EMR for rectal NEN despite achieving complete resection without histologically proven in-depth muscular invasion.

First, regarding indications of endoscopic tumor resection for rectal NEN, NANETS and JNETS guidelines recommend endoscopic resection in cases of a tumor < 10 mm without muscular invasion [7], while ENETS guidelines recommend it in cases of a tumor < 10 mm, G1 or G2, and no muscular invasion [6] because the metastasis rate at diagnosis is reportedly low in 3–9.7% of tumors measuring < 10 mm [4, 5]. On the other hand, endoscopic resection of intermediate 10–20 mm tumors is considered to be of moderate risk despite little evidence [4, 8]. Tumor size > 10 mm, muscular invasion, lymphovascular invasion, mitotic rate > 2/10 high-powered field, and Ki-67 > 2% are reported as the risk factors reflecting liver and lymph node metastasis in the literature [9]. Moreover, Konishi et al. [3] reported that a tumor size > 10 mm is the risk factor of lymph node metastasis and that the prognosis in case of lymph node and distant metastasis is as poor as that of adenocarcinoma; therefore, they recommend surgical resection with lymph node dissection in cases of a tumor size > 10 mm. On the other hand, some reported on the applicability of endoscopic resection in case of intermediate 10–20 mm tumor. In a systematic review, Zhong et al. [10] reported that endoscopic submucosal dissection could be an appropriate treatment choice for tumors smaller than 16 mm in diameter due to the low metastasis rate. Shigeta et al. [11] reported local resection for the high-risk group with a tumor size > 10 mm, that positive lymphovascular invasion developed recurrence in only 1 (4%) of 24 patients (median observation period, 55 months), and that there was no difference in the recurrence rates of high-risk patients who underwent local resection and those who underwent radical resection. They concluded that local resection could be an appropriate treatment choice for tumors measuring 10–20 mm. Thus, appropriate criteria to indicate endoscopic resection are quite controversial. In our three cases, the pathological findings revealed that all tumors were limited to the submucosa and tumor size was 10–13 mm (Table 1). Therefore, EMR was considered retrospectively acceptable according to the expanded indication of endoscopic resection in the recent NANETS and ENETS guidelines.

**Table 1:** Summary of pathological findings of the primary tumor and recurrence

Case no.	Age/sex	Tumor size (mm)	Depth	Margin	Lymphovascular invasion	Ki-67	Recurrence site (Ki-67)	Relapse-free survival (years)
No. 1	55/female	13	Submucosa	Negative	Negative	< 1% (G1)	Liver (8.4%)	10
No. 2	59/female	10	Submucosa	Negative	Negative	5% (G2)	Liver and rectum (5%)	9
No. 3	54/male	12	Submucosa	Negative	Positive	1.7% (G1)	Liver (10%)	13

No consensus was reached on the indications for additional resection after endoscopic resection. The JNETS guideline 2019 highly recommends surgical resection with lymph node dissection as additional resection in the cases that pathological findings indicate lymphovascular invasion, muscular invasion, positive resection margin, and G2 or more, according to the risk factors of lymph node metastasis. ENETS guidelines suggest 6 months of follow-up in cases of G1 with incomplete resection and recommend complete local resection for G2 [6]. On the other hand, Sekiguchi et al. [8] reported no recurrence in cases of positive or negative lymphovascular invasion detected by CD31/synaptophysin double staining and elastic staining; therefore, they insisted that lymphovascular invasion is not a risk factor of recurrence after endoscopic resection. However, their study did not include a long-term follow-up period (median observation time, 67.5 months). In our report, cases 2 and 3 had risk factors for lymph node and liver metastasis described above in terms of Ki-67 (> 2%, case 2) and positive vascular invasion (case 3). There was the potential risk that positive vascular margins caused the liver metastasis in case 3. In our pathological findings, cases 1 and 2 had no lymphovascular invasion. Sekiguchi et al. [8] also revealed vascular invasion was detected by CD31/synaptophysin double staining and elastic staining in 38.9% compared with HE staining alone in only 1%. Thus, it was possible that the prevalence of vascular invasion was underestimated in cases 1 and 2.

Little has been reported about recurrence after long-term follow-up of endoscopic resection for rectal NEN. The five existing reports with long-term follow-up after endoscopic resection are summarized in Table 2. Sekiguchi et al. [8] and Onozato et al. [12] reported no recurrence after endoscopic resection; however, both reports had follow-up periods that were shorter than 7 years. On the other hand, Kwaan et al. [13], Kobayashi et al. [14], and Shigeta et al. [11] reported local recurrence or liver metastasis; however, these reports were cases with positive margins, muscular invasion, or relatively large tumor size. Thus, it is possible that the treatment plan consisting of endoscopic resection and additional resection was inappropriate. In our report, there were no cases with positive margins, and the tumor size was relatively small (10–13 mm); nevertheless, liver metastases were observed during a long-term follow-up period of 9–13 years after endoscopic resection.

**Table 2:** Existing reports describing follow-up after endoscopic resection

	Number	Median observation period (month)	Recurrence (%)	Recurrence site	Relapse-free survival (years)	Features of recurrent cases
Sekiguchi et al. [8]	86	67	0	–	–	–
Onozato et al. [12]	38	77	0	–	–	–
Shigeta et al. [11]	74	31	1 (1.4)	Local	6	Tumor size 20 mm
Kwaan et al. [13]	46	24	1 (2.1)	Liver	5	Positive resection margin, muscular invasion
Kobayashi et al. [14]	38	43	1 (2.6)	Local	16	Positive resection margin

Finally, no consensus has been reached on follow-up period or modality after endoscopic resection. Accordingly, follow-up period and modality differ among guidelines; moreover, no consensus has been reached among facilities in the same country. Lifelong follow-up is considered necessary in ENETS guidelines, as rectal NEN oncologically grows slowly; recurrence was detected at 13 years in our report (case 3). Our report had the limitation that the percentage of metachronous metastases in all patients who underwent endoscopic resection was not investigated. Following all patients who undergo endoscopic resection is highly expensive; therefore, follow-up is required to carefully select high-risk patients. More evidence is required to determine the follow-up strategy after endoscopic resection for rectal NEN.

## Conclusions

Metachronous metastases may occur from endoscopic resection to a long period thereafter; thus, long-term follow-up is necessary after endoscopic resection for rectal NEN. No consensus has been reached on follow-up period or modality after endoscopic resection for rectal NEN. This report may assist with the determination of follow-up strategy after endoscopic resection for rectal NEN.

## Data Availability

Not applicable

## Abbreviations

CT: Computed tomography
EMR: Endoscopic mucosal resection
ENETS: European Neuroendocrine Tumor Society
FNA: Fine needle aspiration
JNETS: Japanese Neuroendocrine Tumor Society
LAR: Low anterior resection
NANETS: North American Neuroendocrine Tumor Society
NEN: Neuroendocrine neoplasm
RFA: Radiofrequency ablation
SD: Stable disease
SSTR-2: Somatostatin-2 receptor
